# Local Diffusion Homogeneity Provides Supplementary Information in T2DM-Related WM Microstructural Abnormality Detection

**DOI:** 10.3389/fnins.2019.00063

**Published:** 2019-02-07

**Authors:** Yi Liang, Han Zhang, Xin Tan, Jiarui Liu, Chunhong Qin, Hui Zeng, Yanting Zheng, Yujie Liu, Jingxian Chen, Xi Leng, Shijun Qiu, Dinggang Shen

**Affiliations:** ^1^Medical Imaging Research Office, Guangzhou University of Chinese Medicine, Guangzhou, China; ^2^Department of Medical Imaging, The First Affiliated Hospital of Guangzhou University of Chinese Medicine, Guangzhou, China; ^3^Department of Radiology and BRIC, University of North Carolina at Chapel Hill, Chapel Hill, NC, United States; ^4^Department of Brain and Cognitive Engineering, Korea University, Seoul, South Korea

**Keywords:** type 2 diabetes mellitus, diffusion tensor imaging, local diffusion homogeneity, white matter, fractional anisotropy

## Abstract

**Objectives:** We aimed to investigate whether an *inter-voxel* diffusivity metric (local diffusion homogeneity, LDH), can provide supplementary information to traditional intra-voxel metrics (i.e., fractional anisotropy, FA) in white matter (WM) abnormality detection for type 2 diabetes mellitus (T2DM).

**Methods:** Diffusion tensor imaging was acquired from 34 T2DM patients and 32 healthy controls. Voxel-based group-difference comparisons based on LDH and FA, as well as the association between the diffusion metrics and T2DM risk factors [i.e., body mass index (BMI) and systolic blood pressure (SBP)], were conducted, with age, gender and education level controlled.

**Results:** Compared to the controls, T2DM patients had higher LDH in the pons and left temporal pole, as well as lower FA in the left superior corona radiation (*p* < 0.05, corrected). In T2DM, there were several overlapping WM areas associated with BMI as revealed by both LDH and FA, including right temporal lobe and left inferior parietal lobe; but the unique areas revealed only by using LDH included left inferior temporal lobe, right supramarginal gyrus, left pre- and post-central gyrus (at the semiovale center), and right superior radiation. Overlapping WM areas that associated with SBP were found with both LDH and FA, including right temporal pole, bilateral orbitofrontal area (rectus gyrus), the media cingulum bundle, and the right cerebellum crus I. However, the unique areas revealed only by LDH included right inferior temporal lobe, right inferior occipital lobe, and splenium of corpus callosum.

**Conclusion:** Inter- and intra-voxel diffusivity metrics may have different sensitivity in the detection of T2DM-related WM abnormality. We suggested that LDH could provide supplementary information and reveal additional underlying brain changes due to diabetes.

## Introduction

Type 2 diabetes mellitus, a complex metabolic disorder characterized by increased blood glucose level, affects more than 425 million people, especially those younger than 65 years (International Diabetes Federation, 2017). T2DM patients could develop many severe complications, among which the increased risk of dementia has been more and more reported (Biessels et al., 2006; Kodl and Seaquist, 2008; McCrimmon et al., 2012). The deterioration of normal frontal lobe functions are frequently reported in T2DM studies (Kawamura et al., 2012; McCrimmon et al., 2012), possibly responsible to cognitive impairment, and malfunctioned executive control abilities (Xia et al., 2013; Cui et al., 2014; Garcia-Casares et al., 2014; Zhang et al., 2014). Since the dementia progression cannot be reversed and a heavy social burden could be elicited, it is of great important to identify potential image-based alterations for better understanding of the cognitive decline in T2DM (Hsu et al., 2012; Biessels and Reijmer, 2014; Brundel et al., 2014; Xia et al., 2017).

Recent evidence based on *in vivo* neuroimaging technique showed that the abnormal neural activities were even found in the T2DM subjects who still have normal cognition (Yang et al., 2016; Zhang et al., 2018). To this end, blood-oxygen-level-dependent (BOLD), functional magnetic resonance imaging (fMRI) has been used as a non-invasive brain functional imaging technique in several T2DM brain functional studies (Brundel et al., 2014). It was reported that the functional connectivity of the default mode network and executive control network in T2DM patients has been impaired despite no clinically significant cognitive decline was found (Yang et al., 2016). However, the neuropathology and pathophysiology of the early cognitive dysfunctions in T2DM are still not clear (Biessels et al., 2006; Kodl and Seaquist, 2008; Kawamura et al., 2012).

Another widely used complementary non-invasive MRI technique is diffusion tensor imaging (DTI), which has higher resolution and better signal-to-noise ratio than fMRI and can sensitively detect changes of constrained molecular water diffusivity in the WM (Alexander et al., 2007). DTI has long been used as a sensitive and objective technique searching for subtle changes in WM in many diseases (O’Donnell and Westin, 2011; Guo W. et al., 2012; Wang et al., 2015; Ding et al., 2018). It is reasonable that WM changes in specific fiber tracts might have led to disrupted information exchange, thus deteriorating functional integration among brain regions (Wakana et al., 2004; Alexander et al., 2007; Reijmer et al., 2013b; Biessels and Reijmer, 2014; Moheet et al., 2015) and further causing cognitive deficits in T2DM. However, for the T2DM patients who have not developed substantial cognitive impairment, only very few DTI studies have been published (Hsu et al., 2012; Zhang et al., 2014), most of which used conventional metrics such as fractional anisotropy (FA) (Beaulieu, 2002; O’Donnell and Westin, 2011; Guo W.B. et al., 2012). One of the limitations of the conventional DTI metrics is that they may not be adequately sensitive to the subtle changes in T2DM patients at the preclinical stage. For example, in a study by our group, we found significantly decreased FA in neurotypical, middle-aged T2DM patients in the fronto-cingulo-parietal areas, cerebellum vermis, and bilateral thalamus (Tan et al., 2016). However, different results have also been reported in other studies (Hsu et al., 2012; Zhang et al., 2014). It might be useful to use another (more sensitive) DTI metrics for T2DM studies.

From the method point of view, FA, and other conventionally used *intra-voxel* diffusivity indices all depend on an assumed tensor model (Alexander et al., 2007), while different models could lead to different results (O’Donnell and Westin, 2011). In a seminal paper (Gong, 2013), an *inter-voxel* measurement, local diffusion homogeneity (LDH), was proposed to reveal more comprehensive WM changes. LDH is a model-free diffusivity index calculated from raw diffusion-weighted images (DWI) that measures the inter-voxel similarity of the full diffusion profiles across a few closely located voxels. Specifically, Kendall’s coefficient of concordance is used to quantify such an overall diffusivity similarity between a centered voxel and those of all its nearest neighborhood (Gong, 2013). For example, a higher LDH value may indicate local coherence enhancement of the fibers, possibly caused by changes in fiber myelination, diameter, or density (Gong, 2013; Liu et al., 2016, 2017).

In this study, we used LDH to study T2DM-related brain changes as helpful supplements to the previous FA-based studies, aiming to test whether it is highly feasible to use such an inter-voxel diffusivity metric in the assessment of T2DM-induced brain WM changes. The LDH- and FA-based results were systematically compared. In addition to the group difference comparisons, we also searched for possible associations between imaging phenotypes (LDH/FA) and certain clinical risk factors (BMI and SBP) for both T2DMs and healthy controls as another evidence of the systematic differences between inter- (LDH) and intra-voxel indices (FA). Our hypothesis is that LDH could detect additional WM alterations compared to FA, which might provide new insights into the neuropathology and pathophysiology underlying the cognitive dysfunction in T2DM.

## Materials and Methods

### Participants

We focused on the T2DM patients without clinically significant cognitive decline or any significant brain diseases in the aim of searching for an early sign of the WM changes. This study was approved by the local ethics committee. Written informed consents from all participants were obtained. T2DM subjects were selected from hospitalized patients from the endocrinology department of the hospital, and healthy controls were from the volunteers over the same period. T2DM was diagnosed using fasting blood glucose >7.0 mmol/L on two separate occasions, or 2-h blood glucose level >11.1 mmol/L during a 75 g oral glucose tolerance test (American Diabetes, 2010; International Diabetes Federation, 2017). All the T2DM subjects received insulin via a pump or subcutaneous injection during hospitalization. All participants received a detailed neurological examination by experienced neurologists to make sure there were no significant cognitive complaints nor positive neurological symptoms. General clinical measurements and demographic information for each subject were collected, including biological tests, chest X-ray, electrocardiogram, BMI [weight (kg)/height (meter)^2^], education level, blood pressure during rest, and duration of the disease (for T2DM patients only).

Exclusion criteria for both groups were as follows: impaired glucose tolerance or impaired fasting glucose (International Diabetes Federation, 2017), serious eye diseases, any sign of cognitive impairment or positive neurological symptoms, any history of neurologic abnormality, serious head injury (with loss of consciousness >5min), severe hypoglycemia or hyperlipemia, left or mixed-handedness, BMI > 30 kg/m^2^, substance (e.g., alcohol, tobacco, psychoactive drug) abuse, hypertension (the cut-off values are based on the Seventh Report of the Joint National Committee on Prevention, Detection, Evaluation, and Treatment of High Blood Pressure (JNC 7), i.e., SBP = 140 mm Hg or a diastolic blood pressure = 90 mm Hg) (Whelton and Carey, 2017), hyperlipemia, specific abnormalities finding in conventional MRI scans, or other factors that might affect brain structure and function (e.g., chronic infections, organic failure, and psychiatric diseases). In addition, all the T2DM subjects took single-field fundus photography for the evaluation of diabetic retinopathy. Based on the International Clinical Disease Severity Scale for diabetic retinopathy, the subjects at the first stage (no apparent retinopathy) and the second stage (mild non-proliferative retinopathy) were included, while those at the third or higher stages, or with macular edema were excluded (Abbas et al., 2017).

### Microvascular Disease Assessment

Small vascular disease [white matter hyperintensities (WMHs) or lacunar infarction] is commonly found in T2DM patients, especial the more elderly patients, and are also associated with neurological impairment (Kodl and Seaquist, 2008). Therefore, it is necessary to make sure these complications will not contribute to our results. WMHs and lacunar infarcts were quantitatively assessed using an age-related WM changes scale (ARWMCs) as described before (Wahlund et al., 2001). Two experienced radiologists blinded to group allocations separately performed the rating. The consensus was obtained through discussion between the two raters if they rated differently. All participants with lacunar infarcts or a rating score >2 were excluded.

### MRI Acquisition

All participants received whole-brain MRI scans with a 3T scanner (SIGNA EXCITE GE Medical Systems, United States) and an 8-channel head coil. The scan time was within 1 week after enrollment and 2–3 h after a meal. First, the axial T1-weighted [repetition time (TR)/echo time (TE) = 2100/24 ms, field of view (FOV) = 22 cm × 22 cm, slice thickness = 5 mm with 1-mm gap], T2-weighted (TR/TE) = 4917/107 ms, FOV = 22 cm × 22 cm, slice thickness = 5 mm with 1-mm gap), and fluid-attenuated inversion recovery (TR/TE = 9000/120 ms, FOV = 24 cm × 24 cm, matrix size = 512 × 512, slice thickness = 5 mm with 1.5-mm gap) were scanned. These clinical scans were used for clinical evaluation to make sure there was no positive finding (e.g., infarction and malformations) from any subject. The high-resolution structural 3D T1-weighted images (256 × 256 image matrix with 160 continuous sagittal slices, 1-mm isotropic voxels) and DTI data (single-shot echo-planar imaging sequence, TR/TE = 12,000/75.5 ms, flip angle = 90°, FOV = 24 cm × 24 cm, matrix size = 128 × 128, axial slice thickness = 3 mm without gap, 25 optional non-linearly distributed directions with b values of 1000 s/mm^2^ and one image with *b* = 0 s/mm^2^, acquisition time = 5 min 36 s) were acquired.

### Image Processing

All DTI data processing was implemented using a pipelined toolbox, PANDA^1^ (Cui et al., 2013), which is based on the FSL^2^ preprocessing pipeline. Eddy-current induced geometric distortions and head motion of all raw diffusion data were firstly corrected. The diffusivity along each diffusion-weighted gradient direction was calculated from the original DWI, which generated a series of diffusivity values at each voxel. Kendall’s coefficient of concordance was applied to quantify the overall similarity of the diffusivity vectors between a centering voxel and its 26 neighborhoods and was attributed to this centering voxel as LDH (Gong, 2013). An LDH map was thus computed for each subject inside a WM mask that was generated by averaging the un-smoothed FA maps across all healthy controls followed by a threshold of FA > 0.25. For comparison, we also generated FA maps for each subject by fitting a diffusion tensor model for each voxel and calculating three eigenvalues (λ1, λ2, and λ3) for voxel-wise FA calculation. For each subject, both LDH and FA maps were generated in the native space. The individual FA maps were non-linearly registered to an FA template in FSL using FNIRT, and the deformation field was applied to each individual’s LDH map to warp them into the common standard space. The LDH and FA maps were further spatially smoothed with a 6-mm full-width-at-half-maximum (FWHM) isotropic Gaussian kernel.

### Group Comparison of Inter- and Intra-Voxel Metrics

To identify the potential T2DM imaging markers based on both LDH and FA, voxel-wise group comparisons between the T2DM subjects and healthy controls within the WM mask were conducted using two-sample *t*-tests for the LDH and FA maps, separately. The two-sample *t*-tests were conducted based on a general linear model with REST v1.8 toolbox (Song et al., 2011). Age, gender, and education level were considered as covariates during the group comparisons and were regressed out. All the statistical maps were corrected for multiple comparisons using a Monte Carlo simulation (AlphaSim), with parameters as follows: FWHM = 6 mm, 1000 simulations, and edge connection. Of note, there are alternative methods to conduct AlphaSim correlation with estimated smoothness from residual maps or statistical maps, which has been suggested as a standard when reporting fMRI results (Poldrack et al., 2008). A voxels level *p* value < 0.01 (two-tailed) and a cluster-level *p*-value < 0.05 with cluster size > 71 mm^3^ were considered to be significant.

### Correlation Between Diffusion Indices and Clinical Measurements

Overweight or obesity (commonly detected by BMI) was an independent risk factor for diabetes, and hypertension (measured by SBP cut points) have also been associated with increased risk of cognitive and cerebrovascular dysfunction (Biessels et al., 2006; Kodl and Seaquist, 2008; Kawamura et al., 2012). Hence, both BMI and SBP were chosen to measure the biological/clinical correlation with both diffusion indices within the T2DM group and control group, separately. Specifically, voxel-wise Pearson’s correction analysis was used to calculate the correlations between diffusion matrixes (LDH and FA) and clinical measurements (BMI and SBP) in the same WM mask. These correlation analyses were also conducted in a voxel-wise manner based on a general linear model with REST v1.8 with the same nuisance covariates added and the same threshold strategy applied (AlphaSim corrected *p* value < 0.05). Such image–clinical measurement association analyses can also demonstrate different sensitivity between these two diffusion indices.

## Results

### Demographic and Clinical Characteristics

Demographic and clinical measurements of the 34 T2DM and 32 controls are summarized in Table 1. T2DM subjects had higher BMI, SBP, education level, and averaged fasting glucose than the healthy controls. Twenty (58.8%) and one (3%) T2DM subject were considered as overweight (BMI, 24–28 kg/m^2^) and obesity (BMI > 28 kg/m^2^), respectively (Shang et al., 2013). Nine (26.5%) T2DM subjects with SBP greater than 130 mmHg were categorized as stage-1 hypertension. No control subject was found to have either a BMI > 24 kg/m^2^ or a SBP > 130 mmHg.

**Table 1:** Demographic and clinical characteristics and diffusion metrics in the regions of interest.

	Controls (*n* = 32)	T2DM (*n* = 34)	*p*
Age (years)	56.31 ± 4.46	58.29 ± 4.19	0.067
Gender (M/F)	18/14	10/24	0.027
BMI (kg/m^2^)	20.58 ± 1.47	24.36 ± 1.89	<0.001
SBP (mmHg)	114.94 ± 5.65	124.38 ± 10.25	<0.001
Diastolic (mmHg)	69.21 ± 4.71	76.26 ± 6.65	0.03
Education (years)	9.75 ± 1.32	10.50 ± 1.21	0.019
FDG (mmol/L)	5.28 ± 0.23	6.64 ± 2.00	<0.001
TG (mmol/L)	1.46 ± 0.30	1.62 ± 0.44	0.101
TC (mmol/L)	4.54 ± 0.36	4.78 ± 0.98	0.205
HDL (mmol/L)	0.96 ± 0.12	1.01 ± 0.19	0.228
LDL (mmol/L)	2.46 ± 0.21	2.55 ± 0.47	0.267
HbA1c (%)	-	7.85 ± 1.34	-
Course of disease (y)	-	6.85 (3-14)	-
LDH at pons	0.48 ± 0.09	0.56 ± 0.08	0.001
LDH at L temporal pole	0.60 ± 0.05	0.64 ± 0.04	0.003
FA at L corona	0.42 ± 0.02	0.39 ± 0.03	<0.001

*Data are presented as means ± SD, numbers of male and female, or median (range). BMI, body mass index; SBP, systolic blood pressure; FDG, averaged fasting glucose; TG, triglyceride; TC, total cholesterol; HDL, high-density lipoprotein; LDL, low-density lipoprotein; HbA1c, glycosylated hemoglobin. LDH at pons, LDH at L temporal pole: LDH values extracted from pons and left temporal pole, respectively; FA at L corona, FA value extracted from left superior corona radiate*.

### Microvascular Disease Assessment

Most T2DM (*n* = 26) and control subjects (*n* = 28) had an ARWMCs scale of 0, while the remaining (eight subjects in T2DM and four in the control group) who were detected with WMHs ≥ 5 mm based on both T2 and FLAIR images had an ARWMCs scale of 1, involving bilateral frontal lobes (*n* = 4), superior radiation (*n* = 6), occipital lobes (*n* = 4) and left temporal lobe (*n* = 1). Lacunar infarction was not found in any of the participants. Four subjects in the T2DM group were classified as the second stage (mild-to-moderate, non-proliferative) retinopathy, and the rest had no apparent retinopathy (American Diabetes, 2010).

### Group Differences in LDH and FA

**Figure 1:**
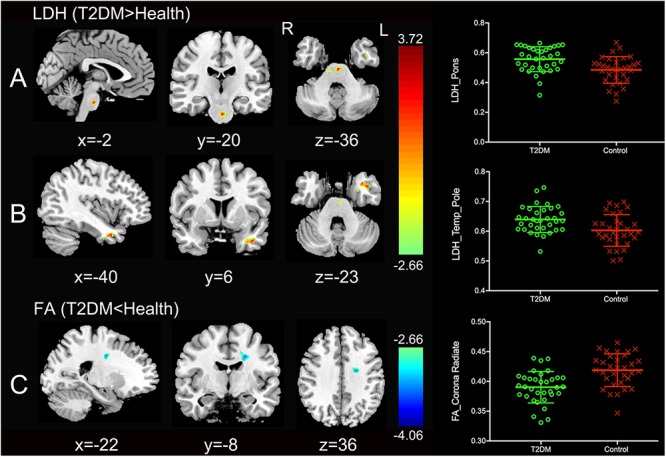
Clusters of between-group differences of LDH and FA with age, gender and education level adjusted (*p* < 0.05, AlphaSim corrected). Significantly higher regional LDH were found in the pons **(A)** and the left temporal pole **(B)**, and significantly decreased FA were found in the left superior corona radiate **(C)** in T2DM subjects compared to the healthy controls. Color scale denotes the *t* values; x, y, z, Montreal Neurological Institutes coordinates; L, left; R, right. The scatter plots show LDH or FA values (means and SD) extracted from each region of interest (ROI) for each group.

**S**ignificantly higher LDH were found in the pons and the left temporal pole in T2DM subjects compared to the healthy controls (Figure 1A,B). There was no decreased LDH observed in the T2DM group. Decreased FA in the T2DM group was only found in the left superior corona radiate (Figure 1C). The mean LDH and FA values within these ROIs were listed in Table 1 and clusters details were listed in Table 2.

**Table 2:** Regions showing group differences in LDH and FA.

Region names (involved metrics)	Voxel size	MNI coordinates (mm)	Peak *T*
Pons (LDH)	109	-2 -20 -36	3.357
L Temporal Pole (LDH)	72	-40 6 -23	3.364
L Corona (FA)	82	-22 -8 36	-3.363

*BA, brodmann area; LDH, local diffusion homogeneity; FA, fractional anisotropy; MCP, the middle cerebellar peduncle; CST, corticospinal tract; UF, uncinate fasciculus; ILF, inferior longitudinal fasciculus; SLF, superior longitudinal fasciculus*.

### Correlation Between Diffusion Indices and Clinical Measurements

**Figure 2:**
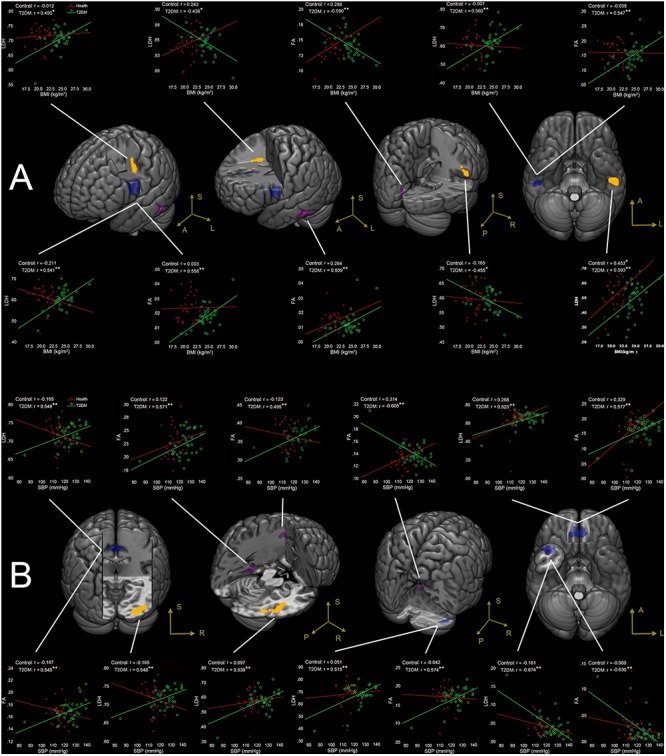
**(A)** Correlations between BMI and LDH/FA, and **(B)** correlations between SBP and LDH/FA in T2DM subjects, with age, gender, and education level adjusted (*p* < 0.05, AlphaSim corrected). The yellow colored clusters show regions affected by BMI and SBP detected only by LDH. The violet colored clusters show regions affected by BMI and SBP only detected by FA. The blue colored clusters show regions affected by BMI and SBP detected by both LDH and FA. The scatter plots show the correlations between clinical measurement (BMI/SBP) and diffusion metrics (LDH/FA) for each cluster and each group. ^∗^*p* < 0.05, ^∗∗^*p* < 0.01. Due to very similar spatial patterns between the two overlapping correlation results, we only show the voxels with significant correlations to LDH to avoid too complex patterns and muddledness in the figure.

No correlations were found between the clinical measures and the LDH/FA within the regions that showed the difference between the two groups. In the following exploratory voxel-wise correlation analysis, BMI- or SBP-associated LDH and FA in the WM were detected for both groups. We found that for the T2DM group, several clusters that presented correlations between BMI/SBP and LDH had similar locations with comparable sizes and peak values to those clusters that presented correlations between BMI/SBP and FA (Table 3, see also the blue-colored areas in Figure 2, which indicated the overlapping clusters using the LDH-based results). From the scatter plots in Figure 2, we found that increased LDH and FA in most of the regions were associated with greater BMI or SBP (*p* < 0.05, corrected). None of these correlations between diffusion metrics and clinical measurements were found in the healthy controls (*p* > 0.05), except the left temporal lobe, where increased LDH values were associated with lower BMI in both groups. All these results were obtained after adjusting for age, gender and education level. Further details are listed in Table 3.

**Table 3:** Regions showing correlations between LDH/FA and BMI/SBP in T2DM.

	Region names	Voxel size	MNI coordinates (mm)	*r* value
BMI & LDH	R inferior temporal lobe (fusiform)^∗1^	136	48 -26 -22	0.542
	L inferior temporal lobe (fusiform)	155	-52 -20 -28	0.592
	R supra-marginal gyrus	139	58 -14 30	-0.606
	L inferior parietal lobe (fusiform)^∗2^	457	-46 8 36	0.661
	R superior radiation	75	30 0 24	-0.574
	L semiovale center/post-central gyrus	157	-44 -10 42	0.573
BMI & FA	R inferior temporal lobe (fusiform)^∗1^	117	50 -24 -28	0.571
	L inferior & middle temporal lobe	104	-68 -34 -18	0.641
	L calcarine cortex (V1)	77	-2 -88 4	-0.714
	L inferior parietal lobe^∗2^	338	-62 6 18	0.629
SBP & LDH	R temporal Pole^&1^	641	48 20 -30	-0.715
	R cerebellum_crus1^&2^	112	42 -68 -28	0.615
	R inferior temporal love	247	48 -40 -20	0.646
	B orbitofrontal area^&3^	276	12 40 -18	0.635
	R inferior Occipital lobe	171	30 -88 -4	0.659
	B cingulum bundle^&4^	256	6 -24 46	0.616
SBP & FA	R cerebellum_Crus1^&2^	136	46 -62 -34	0.620
	R temporal pole^&2^	343	40 22 -34	-0.664
	B orbitofrontal area^&3^	206	-2 40 -22	0.674
	L parahippocampus	168	-20 -44 -8	0.670
	Vermis	110	2 -56 2	-0.669
	L superior radiation	111	-26 8 34	0.546
	B cingulum bundle^&4^	71	6 -26 44	0.634

*BA, brodmann area; V1, primary visual cortex; OR, optic radiations; LDH, local diffusion homogeneity; FA, fractional anisotropy; BMI, body mass index; SBP, systolic blood pressure; UF, uncinate fasciculus; ILF, inferior longitudinal fasciculus; SLF, superior longitudinal fasciculus; FPUT, the fronto-parietal U-tracts connecting pre-central and post-central gyrus; CST, corticospinal tract; IFO, inferior fronto-occipital fasciculus; sCC, the fibers of splenium of corpus callosum; mCB, medial cingulum bundle; gCC, genu of corpus callosum; FX, fornix; ST, stria terminalis; R, right, L, left; B, bilateral. ^∗^Overlapping regions associated with BMI as revealed by both LDH and FA. ^&^Overlapping regions associated with SBP as revealed by both LDH and FA. The number behind ^∗^ or ^&^: the pairing label for each overlapping region that presents the correlation between LDH/FA and BMI, or between LDH/FA and SBP*.

#### BMI-Associated WM Changes

Four clusters (the yellow clusters in Figure 2A) presented BMI-associated WM changes *only* by using LDH, including left inferior temporal lobe, right supra-marginal gyrus; left pre-central gyrus and post-central gyrus (semiovale center), and right superior radiation. Two clusters (the violet clusters in Figure 2A) presented BMI-associated WM changes only by using FA, including the left inferior temporal lobe, and left calcarine cortex. There were two marked overlapping WM areas associated with BMI as revealed by both LDH and FA, including the right inferior temporal lobe and left inferior parietal lobe (the blue clusters in Figure 2A).

#### SBP-Associated WM Changes

Likewise, SBP-associated WM regions using only LDH are shown in Figure 2B (the yellow blobs), including the right inferior temporal lobe, the right inferior occipital lobe, and the splenium of corpus callosum. The SBP-associated WM regions using only FA located in the left superior corona radiation, the left parahippocampus, and the vermis. The overlapping SBP-associated regions revealed by both LDH and FA included the right temporal pole, bilateral orbitofrontal area (rectus gyrus), the media cingulum bundle, and the right cerebellum crus I (shown in blue in Figure 2B).

## Discussion

### Major Findings and the Clinical Indications

To our best knowledge, this is the first study that comprehensively compare LDH and FA in a imaging-based clinical research, and the first study using LDH for DWI-based brain alteration detection in T2DM (American Diabetes, 2010; Liu et al., 2016, 2017; Zhuo et al., 2016). In this study, voxel-based intra-voxel (FA) and inter-voxel diffusivity metrics (LDH) were used in a whole-brain exploratory study to explore and compare their sensitivity in between-group comparison and brain-clinical association analysis. The results confirmed our hypothesis that (1) inter-voxel and intra-voxel diffusivity metrics had different sensitivity in T2DM-related WM microstructural abnormality detection, and (2) both metrics provided supplementary information to each other in the detection of WM changes for T2DM.

### Group Difference in LDH and FA

#### LDH and FA Are Complementary for T2DM Imaging Marker Detection

We found increased LDH in the T2DM patients in the pons and the left temporal pole, while FA had no such changes. There are two possible reasons lead to such differences. First, LDH and FA are two different diffusion parameters with different sensitivity to WM impairments. LDH measures the *inter-voxel* similarity of the diffusivity profile in a local range, while FA measures *intra-voxel* diffusivity shape (Alexander et al., 2007). It has been speculated that LDH may be *more sensitive* to the microstructural coherence changes but less sensitive to the myelination changes than FA (Gong, 2013). Secondly, the pons and the left temporal pole contain WM tracts of complex nature (e.g., the crossing fibers) (Alexander et al., 2007; Kiernan, 2012). FA can be largely affected by the crossing fibers (Alexander et al., 2007; Gong, 2013). LDH, on the other hand, is a model-free index that has been suggested to be more tolerable to fiber crossing (Gong, 2013; Liu et al., 2016, 2017).

We also found that, in the left corona radiata, there was a group difference in FA but not LDH. This is consistent with previous findings using FA for T2DM imaging marker detection (Tan et al., 2016). Interestingly, the corona radiata also has crossing fibers. Therefore, we assumed that the left corona radiata could have adequate group difference, where the crossing-fiber influence of FA could not cancel out the detection of such a difference (Tan et al., 2016). For LDH, we speculated that this region might have little changes in the fiber orientation coherence, but more possibly subjecting demyelination (Gong, 2013).

#### Increased LDH in Pons and the Left Temporal Pole Reflect Compensatory Effect

Increased LDH indicates the enhancement of coherence of local fibers, which may be due to changes of the fiber myelination, diameter, or density differences along the WM tracts. We interpreted such an LDH increase as consequences of an early compensatory mechanism by neuroplasticity that could eventually disappear as the disease progresses (Gispen and Biessels, 2000; Gong, 2013). The increased LDH in the pons could reflect cerebellar compensation to the impaired cerebral functions, or probably result from the functional enhancement of the corticospinal tract (CST). These compensatory mechanisms can be generally mediated by anatomy pathways via the pons. Specifically, the pons contains fibers connecting the cerebellum (via the middle cerebellar peduncle) and the cerebral cortex (via CST) (Wakana et al., 2004; Buckner, 2013) as a pivotal “relay station” and a connector. Several studies have found the possible compensation mechanisms by the cerebellum in T2DM patients (Xia et al., 2013; Cui et al., 2014; Wang et al., 2017). Although these studies were based on the subjects with different demographic and cognitive characteristics, the differences in the cerebellum could still be detected in all of these studies due to its compensatory ability to maintain intact cognitive functions (Stoodley et al., 2010; Buckner, 2013; Cui et al., 2014). On the other hand, T2DM patients could be easily affected by the diabetic peripheral neuropathy, which could further lead to altered sensory function (Ba-Tin et al., 2011). Moreover, T2DM subjects often have increased BMI or even obesity, which can impair fine motor control and cause movement disorder (Berrigan et al., 2006). All these impaired sensory and/or motor functions could be compensated by the increased LDH in the pons to enhance the CST for maintaining normative neural functions in the preclinical stage (Hsu et al., 2012; van Bloemendaal et al., 2016).

The left temporal pole has strong anatomical connections to paralimbic regions including hippocampus, parahippocampus, amygdala, hypothalamus, and insula (Olson et al., 2007; Kiernan, 2012). It also mediates several high-order cognitive functions, such as memory and emotion processing (Kodl and Seaquist, 2008; Kawamura et al., 2012; McCrimmon et al., 2012). These regions particularly have abundant insulin receptors that are believed to be essential for memory and other cognitive functions (Kiernan, 2012; McCrimmon et al., 2012). In T2DM, hyperinsulinemia or insulin-resistance leads to reduced insulin receptors, which could affect the abovementioned regions and cause impaired cognitive functions (Kamal et al., 1999; van Bloemendaal et al., 2016; Alfaro et al., 2018). Therefore, the increased LDH in the temporal pole could reflect strengthened connections to these cognitive function-related regions and make compensation to maintain normal cognitive functions.

To further explore such a possible compensatory effect, we did a supplementary experiment focusing on WM structural connectivity networks and found reduced local efficiency at the right superior temporal pole (*p* < 0.05 after Bonferroni correction, see Supplementary Material). This finding provides another support for our claim, that is, the increased LDH in the left-sided temporal pole could be caused by reduced local efficiency in its right-sided counterpart. Such a compensatory hypothesis needed to be further investigated by future studies.

### Correlation Between Diffusion Metrics and Clinical Measurements

In the T2DM group, different WM regions were associated with BMI and SBP, irrespective of which diffusion metrics (LDH or FA) was used. These results indicated not only different sensitivity of the two diffusivity metrics but also different impacts of obesity and hypertension on the brain (Biessels et al., 2006; Sery et al., 2014; Kullmann et al., 2015). In general, BMI-associated WM regions in the T2DM group mainly encompassed the bilateral association fibers (i.e., fibers that cross the inferior temporal lobe) and the fibers connecting the motor and somatosensory areas (pre- and post-central gyrus). Meanwhile, the SBP-associated WM regions in the T2DM group were more widely distributed, including fibers in the limbic system (left parahippocampus and the media cingulum bundle), association fibers extending to the temporal lobe, callosal fibers, and cerebellar WM (vermis and right cerebellum crus I). The current study showed consistent results with previous research (Hassing et al., 2004; Brundel et al., 2014; van Bloemendaal et al., 2016; Alfaro et al., 2018). For example, it has been reported that the significant alterations of odor-induced brain activations, especially the significantly decreased activation in the hippocampus and parahippocampus of the dominant hemisphere could occur before brain structural changes in T2DM with normal cognitive functions (Zhang et al., 2018). We noticed that in the current study, increased LDH and FA values in most of the regions (e.g., bilateral orbitofrontal area and the left parahippocampus) were associated with greater BMI or SBP; while inverse correlations between the clinical measurements and the diffusion metrics were mainly in the right hemisphere. Since greater BMI and SBP were associated with increased risks of cognitive dysfunctions (Hassing et al., 2004; Alfaro et al., 2018), the inverse brain-clinical associations in our study suggest potential damage of WM fiber structure in T2DM even before the onset of symptoms. Likewise, such brain-clinical associations in the opposite direction could also be explained as recruitment capacity to maintain the cognitive performance in circumstances of altered BMI or SBP (Biessels et al., 2006; Liu et al., 2017; Xia et al., 2017).

The mechanisms of how obesity and hypertension affect the human brains WM integrity have not been fully elucidated, because the T2DM *per se* and its metabolic syndromes could share common pathways, which lead to complex metabolic, inflammatory and microvascular disturbances (van Bloemendaal et al., 2016; Alfaro et al., 2018). All these factors may weigh into WM microstructural damages and cognitive decline; thus, it is difficult to differentiate the biological and neurological consequences of each individual factor (Biessels et al., 2006; Toth et al., 2006; Geha et al., 2017; Alfaro et al., 2018). Taken together, we propose that the correlations between brain imaging metrics and clinical measurements could be a probable result from brain plasticity and other compensatory mechanisms, which allow the brain to adapt according to the changes in environmental pressure, physiology, and pathology caused by T2DM in order to maintain a normative level of cognitive functions (Kamal et al., 1999; van Harten et al., 2006; Biessels and Reijmer, 2014; Concha, 2014; van Bloemendaal et al., 2016; van Bussel et al., 2017).

### Strengths and Limitations

The current study features several strengths. First, to our best knowledge, it is the first to investigate inter-voxel diffusivity metrics in the T2DM brains. It is also the first comprehensive investigation that combines between-group comparisons and a brain-clinical measure association analysis, which together comprehensively reveal the T2DM-related brain alterations. Second, the two groups involved in this study had relative younger age and narrow age range compared to most of the other studies (Reijmer et al., 2013a; van Bussel et al., 2017; Xia et al., 2017). Such features could alleviate the cohort heterogeneity problem and reduce the nuisance effect of age (Gispen and Biessels, 2000; Biessels et al., 2006; Salthouse, 2009). Third, low microvascular risk (i.e., no infarction, no significant WMHs, and no apparent retinopathy) with no apparent cognitive impairments in the two groups could further minimize confounding effects (Peng et al., 2016).

One of the possible concerns is the unmatched education level and gender. However, we could not find out any significant gender- and education level-effect on the LDH and FA from a linear regression test. Meanwhile, all the main analysis were conducted with age, gender and education level as covariates to exclude their potential influences. Therefore, we believe that education level and gender could have an insignificant effect on the results. Due to the difficulty of collecting middle-aged cognitively normal T2DM patients, we intended to include as many subjects as possible in this study. Another limitation is that the true biological meaning of LDH is still unclear by now. Although we have made tentative explanations and interpretations in terms of potential neuromechanism and biological substrates of the LDH, these interpretations are stillhighly speculative. Future studies may need to manipulate the WM changes with a specific disease or animal model to further investigate the neural basis of LDH changes.

## Conclusion

Our study indicated that inter-voxel (local diffusion homogeneity, LDH) and intra-voxel (FA) metrics had different sensitivity in detection of T2DM-related WM microstructural abnormalities. The combination of FA and LDH could provide supplementary information and better reveal the underlying brain changes due to diabetes. We found interesting compensatory recruitment of the pons and the left temporal pole with increased LDH in T2DM compared to the healthy controls. Such a compensatory mechanism and the potential associations between risk factors and imaging findings in the middle-aged T2DM patients hold great clinical potential in detecting early imaging markers of T2DM.

## Ethics Statement

This study was approved by the ethics committee of First Affiliated Hospital of Guangzhou University of Chinese Medicine. All subjects gave written informed consent in accordance with the Declaration of Helsinki.

## Author Contributions

HaZ, SQ, and DS contributed to conception and design of the study. YuL, HuZ, and XT organized the data. YiL performed the data analysis and drafted the manuscript. All authors revised the manuscript, and read and approved the submitted version.

## Conflict of Interest Statement

The authors declare that the research was conducted in the absence of any commercial or financial relationships that could be construed as a potential conflict of interest.

## Footnotes

1 http://www.nitrc.org/projects/panda

2 http://fsl.fmrib.ox.ac.uk/fsl

## Abbreviations

ARWMCs: age-related white matter changes
BMI: body mass index
CST: corticospinal tract
DTI: diffusion tensor image
DWI: diffusion-weighted image
FA: fractional anisotropy
FOV: field of view
LDH: local diffusion homogeneity
MRI: magnetic resonance image
SBP: systolic blood pressure
T2DM: type 2 diabetes mellitus
TE: echo time
TR: repetition time
WM: white matter
WMHs: white matter hyperintensities
