# Supplementary material for: Local Diffusion Homogeneity Provides Supplementary Information in T2DM-Related WM Microstructural Abnormality Detection
Source: Front Neurosci. 2019 Feb 7;13:63. doi: 10.3389/fnins.2019.00063 (PMC6374310; doi:10.3389/fnins.2019.00063)
Supplement: Supplementary file 1 [file Table_1.docx]

Supplementary Material

**Local Diffusion Homogeneity provides supplementary information in T2DM-related WM microstructural abnormality detection**

Yi Liang^1,2 †^, Han Zhang^3 †^, Xin Tan^2^, Jiarui Liu^1^, Chunhong Qin^2^, Hui Zeng^1^, Yanting Zheng^1^, Yujie Liu^1^, Jingxian Chen^1^, Xi, Leng^2^, Shijun Qiu^1, 2^ *, Dinggang Shen^3,4 *^

^1^ Guangzhou University of Chinese medicine, Guangzhou, Guangdong 510405, China

^2^ Department of Medical Imaging, The First Affiliated Hospital of Guangzhou University of Chinese Medicine, Guangzhou, Guangdong, 510405, China

^3^ Department of Radiology and BRIC, University of North Carolina at Chapel Hill, Chapel Hill, NC 27599, USA

^4^ Department of Brain and Cognitive Engineering, Korea University, Seoul 02841, Republic of Korea

† Yi Liang, Han Zhang contributed equally to this work.

* **Corresponding Authors:**

Shijun Qiu, MD, Ph.D.

Email: qiu-sj@163.com

Tel/Fax: +86-20-36585090

Address: 16 Jichang Road, Guangzhou 510405, China

Dinggang Shen, Ph.D.

Email: dgshen@med.unc.edu

Tel: +1 (919) 966-3535

Fax: +1 (919) 843-2641

Address: CB #7513, 130 Mason Farm Road,

Chapel Hill, NC 27599, United States

**Difference in Local Efficiency between T2DMs and Healthy Controls**

This supplementary study showed that, for the middle-aged T2DM patients who had no clinically significant cognitive impairment, reduced *local efficiency* compared to age-, gender- and education level-matched healthy controls were found at the *right* temporal pole. In the main text, we found increased *local diffusion homogeneity* (LDH) in the *left* temporal pole, the mirrored region of the right temporal pole with decreased local efficiency. This may further indicate the proposed *compensatory* *effect* mediated by the abnormally increased LDH in the other side of the temporal pole. That is, increased LDH in the left temporal pole could provide compensatory resources to prevent middle-aged T2DM patients from obvious cognitive impairments.

Table 1 shows the demographic and clinical data of the age-, gender- and education level-matched subjects from the two groups used in this supplementary experiment. To investigate local efficiency at each brain region, the brain structural connectivity network was constructed for each subject, where 116 automated anatomical labeling (AAL) template was used to define nodes and the edges were built if there were white matter tracts interconnecting any pair of the nodes. The weight at each edge was determined by the averaged fractional anisotropy (FA) along the white matter tracts. Brain Connectivity toolbox was used to calculate local efficiency for each brain region from each subject based on the weighted structural connectivity network. Local efficiency reflects the information transmission efficiency of a local cluster centering at each brain region. Two sample t-tests were conducted for between-group comparisons with age, gender, and education level controlled (*p* < 0.05, Bonferroni correction).

Only right superior temporal pole was found to have significantly reduced local efficiency in the T2DM patients compared to the matched controls.

**Supplementary Table 1 Demographic data of the two groups**

|  | T2DM (n=32) | Control (n=33) | *p* |
| --- | --- | --- | --- |
| Age (years) | 57.38 ± 8.81 | 55.91± 9.55 | 0.523 |
| Gender (M/F) | 11/21 | 18/15 | 0.102 |
| Education (years) | 10.22 ± 1.66 | 10.55 ± 1.28 | 0.376 |
| BMI (kg/m^2^) | 24.58 ± 1.98 | 20.81 ± 1.44 | 0.000 |
| SBP (mmHg) | 124.53 ± 8.34 | 114.30 ± 7.12 | 0.000 |
| Diastolic (mmHg) | 69.21 ± 4.71 | 76.26 ± 6.65 | 0.030 |
| FDG (mmol/L) | 6.64 ± 2.00 | 5.28 ± 0.23 | <0.001 |
| TG (mmol/L) | 1.62 ± 0.44 | 1.46 ± 0.30 | 0.101 |
| TC (mmol/L) | 4.78 ± 0.98 | 4.54 ± 0.36 | 0.205 |
| HDL (mmol/L) | 1.01 ± 0.19 | 0.96 ± 0.12 | 0.228 |
| LDL(mmol/L) | 2.55 ± 0.47 | 2.46 ± 0.21 | 0.267 |
| HbA1c (%) | 8.03 ± 1.29 | - | - |
| Duration of disease (years) | 6.85 (3-14) | - | - |

Data are presented as means ± SD, numbers of male and female, or median (range).

BMI, body mass index; SBP, systolic blood pressure; FDG, averaged fasting glucose; TG, triglyceride; TC, total cholesterol; HDL, high density lipoprotein; LDL, low density lipoprotein; HbA1c, glycosylated hemoglobin.
